# Performance of standardised colposcopy to detect cervical precancer and cancer for triage of women testing positive for human papillomavirus: results from the ESTAMPA multicentric screening study

**DOI:** 10.1016/S2214-109X(22)00545-9

**Published:** 2023-02-14

**Authors:** Joan Valls, Armando Baena, Gino Venegas, Marcela Celis, Mauricio González, Carlos Sosa, Jorge Luis Santin, Marina Ortega, Ana Soilán, Elmer Turcios, Jacqueline Figueroa, Margarita Rodríguez de la Peña, Alicia Figueredo, Andrea Verónica Beracochea, Natalia Pérez, Josefina Martínez-Better, Oscar Lora, Julio Yamil Jiménez, Diana Giménez, Laura Fleider, Yuly Salgado, Sandra Martínez, Yenny Bellido-Fuentes, Bettsy Flores, Silvio Tatti, Verónica Villagra, Aurelio Cruz-Valdez, Carolina Terán, Gloria Inés Sánchez, Guillermo Rodríguez, Maria Alejandra Picconi, Annabelle Ferrera, Laura Mendoza, Alejandro Calderón, Raul Murillo, Carolina Wiesner, Nathalie Broutet, Silvana Luciani, Carlos Pérez, Teresa M Darragh, José Jerónimo, Rolando Herrero, Maribel Almonte, ESTAMPA study group

**Affiliations:** a Early Detection, Prevention and Infections Branch, International Agency for Research on Cancer, Lyon, France; b Centro de Investigación Biomédica en Red de Cáncer (CIBERONC), Madrid, Spain; c Clínica Angloamericana, Lima, Peru; d Escuela de Medicina Humana, Universidad de Piura, Lima, Peru; e Instituto Nacional de Cancerología, Bogotá, Colombia; f Hospital Monseñor Victor Manuel Sanabria Martínez, Puntarenas, Costa Rica; g Hospital Nacional, Ministerio de Salud Pública y Bienestar Social, Itauguá, Paraguay; h Instituto Nacional del Cáncer, Ministerio de Salud Pública y Bienestar Social, Capiatá, Paraguay; i Hospital Materno Infantil de San Lorenzo, Ministerio de Salud Pública y Bienestar Social, San Lorenzo, Paraguay; j Programa Nacional contra el Cáncer, Tegucigalpa, Honduras; k Hospital Nacional Profesor Alejandro Posadas, Buenos Aires, Argentina; l Centro de Salud Ciudad de la Costa, Ciudad de la Costa, Uruguay; m Hospital Policial, Montevideo, Uruguay; n Hospital de Clínicas, Facultad de Medicina, Montevideo, Uruguay; o Ese Hospital Antonio Roldán Betancur, Apartadó, Colombia; p Facultad de Medicina, Universidad Mayor, Real y Pontificia de San Francisco Xavier de Chuquisaca, Sucre, Bolivia; q Hospital Gineco-Obstétrico y Neonatal Dr Jaime Sánchez Porcel, Sucre, Bolivia; r Instituto de Salud Pública de Mexico, Morelos, Mexico; s Hospital Materno Infantil de Trinidad, Ministerio de Salud Pública y Bienestar Social, Asunción, Paraguay; t Hospital de Clínicas, José de San Martín, Buenos Aires, Argentina; u Liga contra el Cáncer-Peru, Lima, Peru; v Laboratorio Central de Salud Pública, Asunción, Paraguay; w Grupo de Infección y Cáncer, Universidad de Antioquía, Medellín, Colombia; x Comisión Honoraria de Lucha contra el Cáncer, Montevideo, Uruguay; y Instituto Nacional de Enfermedades Infecciosas—ANLIS Malbrán, Buenos Aires, Argentina; z Instituto de Infecciones en Microbiología, Universidad Nacional Autónoma de Honduras, Tegucigalpa, Honduras; aa Instituto de Investigaciones en Ciencias de la Salud, Universidad Nacional de Asunción, San Lorenzo, Paraguay; ab Caja Costarricense de Seguro Social, Región Pacífico Central, San José, Costa Rica; ac Department of Sexual and Reproductive Health and Research, WHO, Geneva, Switzerland; ad Pan American Health Organization, Washington, DC, USA; ae Department of Pathology, University of California, San Francisco, CA, USA; af US National Cancer Institute, Bethesda, MD, USA; ag Agencia Costarricense de Investigaciones Biomédicas, Fundación Inciensa, Guanacaste, Costa Rica

## Abstract

**Background:**

Colposcopy, currently included in WHO recommendations as an option to triage human papillomavirus (HPV)-positive women, remains as the reference standard to guide both biopsy for confirmation of cervical precancer and cancer and treatment approaches. We aim to evaluate the performance of colposcopy to detect cervical precancer and cancer for triage in HPV-positive women.

**Methods:**

This cross-sectional, multicentric screening study was conducted at 12 centres (including primary and secondary care centres, hospitals, laboratories, and universities) in Latin America (Argentina, Bolivia, Colombia, Costa Rica, Honduras, Mexico, Paraguay, Peru, and Uruguay). Eligible women were aged 30–64 years, sexually active, did not have a history of cervical cancer or treatment for cervical precancer or a hysterectomy, and were not planning to move outside of the study area. Women were screened with HPV DNA testing and cytology. HPV-positive women were referred to colposcopy using a standardised protocol, including biopsy collection of observed lesions, endocervical sampling for transformation zone (TZ) type 3, and treatment as needed. Women with initial normal colposcopy or no high-grade cervical lesions on histology (less than cervical intraepithelial neoplasia [CIN] grade 2) were recalled after 18 months for another HPV test to complete disease ascertainment; HPV-positive women were referred for a second colposcopy with biopsy and treatment as needed. Diagnostic accuracy of colposcopy was assessed by considering a positive test result when the colposcopic impression at the initial colposcopy was positive minor, positive major, or suspected cancer, and was considered negative otherwise. The main study outcome was histologically confirmed CIN3+ (defined as grade 3 or worse) detected at the initial visit or 18-month visit.

**Findings:**

Between Dec 12, 2012, and Dec 3, 2021, 42 502 women were recruited, and 5985 (14·1%) tested positive for HPV. 4499 participants with complete disease ascertainment and follow-up were included in the analysis, with a median age of 40·6 years (IQR 34·7–49·9). CIN3+ was detected in 669 (14·9%) of 4499 women at the initial visit or 18-month visit (3530 [78·5%] negative or CIN1, 300 [6·7%] CIN2, 616 [13·7%] CIN3, and 53 [1·2%] cancers). Sensitivity was 91·2% (95% CI 88·9–93·2) for CIN3+, whereas specificity was 50·1% (48·5–51·8) for less than CIN2 and 47·1% (45·5–48·7) for less than CIN3. Sensitivity for CIN3+ significantly decreased in older women (93·5% [95% CI 91·3–95·3] in those aged 30–49 years *vs* 77·6% [68·6–85·0] in those aged 50–65 years; p<0·0001), whereas specificity for less than CIN2 significantly increased (45·7% [43·8–47·6] *vs* 61·8% [58·7–64·8]; p<0·0001). Sensitivity for CIN3+ was also significantly lower in women with negative cytology than in those with abnormal cytology (p<0·0001).

**Interpretation:**

Colposcopy is accurate for CIN3+ detection in HPV-positive women. These results reflect ESTAMPA efforts in an 18-month follow-up strategy to maximise disease detection with an internationally validated clinical management protocol and regular training, including quality improvement practices. We showed that colposcopy can be optimised with proper standardisation to be used as triage in HPV-positive women.

**Funding:**

WHO; Pan American Health Organization; Union for International Cancer Control; National Cancer Institute (NCI); NCI Center for Global Health; National Agency for the Promotion of Research, Technological Development, and Innovation; NCI of Argentina and Colombia; Caja Costarricense de Seguro Social; National Council for Science and Technology of Paraguay; International Agency for Research on Cancer; and all local collaborative institutions.

## Introduction

More than 600 000 new cases of cervical cancer occur worldwide each year and account for nearly 350 000 deaths. More than 90% of deaths are observed in low-income and middle-income countries (LMICs),^1^ where screening efforts have been suboptimal.

The traditional screening approach based on cytology, colposcopy, histological confirmation, and treatment of cervical disease has been successful at reducing cervical cancer incidence when applied systematically and with high coverage;^2^, ^3^, ^4^ however, this approach has rarely been effective in LMICs. 25 years ago, most concerns regarding the limitations of cervical cancer screening were focused on the variable quality and low sensitivity of cytology.^5^ Over the past decade, human papillomavirus (HPV) testing has been progressively replacing cytology in primary screening. High sensitivity of HPV testing comes at the cost of lower specificity; consequently, a second triage test is usually required to reduce referrals and overtreatment. Current WHO guidelines^6^ recommend using HPV testing for primary screening and suggest either treatment of all HPV-positive women or a screen, triage, and treat approach using partial genotyping, colposcopy, visual inspection with acetic acid, or cytology to triage women after a positive HPV test.

The main goal of colposcopy, first described as a method for early cervical cancer detection more than 90 years ago,^7^ is to detect cervical precancer which can be treated to prevent cervical cancer. Colposcopy includes visual inspection of the cervix using dilute acetic acid, magnification, and a strong light source and remains the standard procedure to guide biopsy and treatment approaches. Despite having a major role in clinical diagnosis, colposcopy should not be used as primary screening.^8^ Current WHO guidelines^6^ do not support its use in this context and screening programmes usually only consider referral to colposcopy after a positive screening test.

**Research in context**

**Evidence before this study**

In the past 30 years, the accuracy of colposcopy to detect cervical precancer and cancer has been studied mainly in women with abnormal cytology results. We searched PubMed for systematic reviews and meta-analyses from database inception to Dec 1, 2021, using the search terms (“colposcopy”[Title]) AND ((“accuracy”) OR (“diagnosis”)), without any language restrictions. We identified four studies that compiled evidence on the accuracy of colposcopy as a diagnostic method for cervical cancer and precancer, showing highly variable sensitivity estimates, ranging from 30% to 100%. However, none focused specifically on colposcopy as triage for human papillomavirus (HPV)-positive women. Current WHO guidelines published in July, 2021, include colposcopy among recommendations as an option of triage for HPV-positive women. These recommendations were supported by a meta-analysis using individual data from three previously published studies in the context of cervical cancer screening, reporting a sensitivity of 86% (95% CI 78–92) and a specificity of 72% (61–83) for the performance of colposcopy to predict cervical intraepithelial neoplasia (CIN) grade 3 or worse in HPV-positive women. Additionally, several studies have investigated heterogeneity in the performance of colposcopy, which can be partly explained by differences in positivity thresholds, disease definitions, detection, and methods used for evaluations. Moreover, factors such as clinician experience, biopsy placement, and the number of biopsies collected can influence colposcopy accuracy and can lead to clinically relevant cervical lesions being missed or patients having to undergo additional unnecessary diagnostic procedures.

**Added value of this study**

To our knowledge, this cross-sectional, multicentre study is the first major evaluation of colposcopy as triage in HPV-positive women. We assessed different triage techniques to detect cervical precancer and cancer in women aged 30–64 years across nine countries in Latin America. HPV-positive women were referred to colposcopy using a standardised protocol that included mandatory biopsy of observed acetowhite lesions, endocervical sampling for transformation zone type 3, and colposcopy-guided treatment. Participants without high-grade disease were tested again for HPV after 18 months, and those testing positive were referred to a second colposcopy with biopsies and treatment as needed. 4499 (11%) of 42 502 recruited women were HPV positive and had complete disease ascertainment, and CIN2+ was detected in 969 (300 with CIN2, 616 with CIN3, and 53 with cancer). Our results show an excellent overall performance of colposcopy with a 91·2% sensitivity for CIN3+ detection in HPV-positive women (90·4% sensitivity for CIN2+), a high positive predictive value (23·1%) compared with the proportion of CIN3+ detected among all HPV-positive women (14·9%), and an acceptable specificity (50·1%) in the context of triage.

**Implications of all the available evidence**

Colposcopy can be optimised with appropriate standardisation to effectively triage for HPV-positive women. In the context of the 2030 WHO global strategy to accelerate the elimination of cervical cancer, our results reinforce WHO recommendations on the use of colposcopy as triage for HPV-positive women. Our findings not only contribute to improving the clinical management of HPV-positive women screened in different scenarios, but also might lead to the revision of colposcopy clinical practice guidelines worldwide.

Colposcopy has mainly been used to evaluate women referred after an abnormal cytology result. For this indication, colposcopy has shown heterogeneous sensitivity in different clinical settings, with previous meta-analyses reporting highly variable sensitivity estimates (ranging from 30% to 100%).^9^, ^10^, ^11^, ^12^ Factors such as clinician experience, biopsy placement, and the number of biopsies collected can influence the accuracy of colposcopy to detect cervical intraepithelial neoplasia (CIN) grade 2 and CIN grade 3 lesions^13^, ^14^ and lead to clinically relevant cervical lesions being missed or additional unnecessary diagnostic procedures. The choice of where to biopsy and whether to collect one or more biopsies might be more important than assigning a colposcopic impression.^15^

ESTAMPA aims to evaluate the performance of different screening and triage techniques to prevent cervical cancer and to inform the implementation of HPV-based cervical cancer screening programmes in LMICs. Here, we aim to evaluate the performance of colposcopy as triage to detect cervical precancer and cancer among HPV-positive women.

## Methods

### Study design and participants

This cross-sectional, multicentric screening study was conducted at 12 centres (including primary and secondary care centres, hospitals, laboratories, and universities) in nine countries (Argentina, Bolivia, Colombia, Costa Rica, Honduras, Mexico, Paraguay, Peru, and Uruguay) within Latin America. Details of the study design, methods, and protocol are published elsewhere.^16^ Eligible women were aged 30–64 years, sexually active, did not have a history of cervical cancer or treatment for cervical precancer or a hysterectomy, and were not planning to move outside of the study area. Women were screened with HPV DNA testing and cytology. For this analysis, only those who tested positive for HPV were included. HPV-positive women were referred to colposcopy for disease ascertainment and treatment, as needed. Women with initial normal colposcopy or no high-grade cervical lesions on histology (less than CIN2) were recalled after 18 months for another HPV test to complete disease ascertainment; those who tested HPV positive again were referred for a second colposcopy with biopsy and treatment as needed. All CIN2+ (defined as grade 2 or worse) lesions were treated appropriately.

The protocol was approved by ethics committees of the WHO International Agency for Research on Cancer (project 12–27-A7) and the Pan American Health Organization, and those at study centres. All participants provided written informed consent. This study is registered with ClinicalTrials.gov (NCT01881659).

### Procedures

#### HPV testing and cytology

During the initial screening visit, exfoliative cervical samples were taken with a Cervex-Brush (Rovers Medical Devices, Oss, Netherlands) and used for conventional cytology, which was classified according to the Bethesda System as negative, atypical squamous cells of undetermined signiﬁcance (ASC-US), low-grade squamous intraepithelial lesion (LSIL), and high-grade squamous intraepithelial lesion (HSIL). Immediately after cytology, the residual cells remaining on the brush were rinsed into vials containing 20 mL of preservative ThinPrep PreservCyt medium (Hologic, Marlborough, MA, USA) for HPV testing. HPV testing was performed according to manufacturer's instructions at independent laboratories using the digene HC2 High-Risk HPV DNA Test (QIAGEN, Germantown, MD, USA) or COBAS 4800 HPV Test (Roche Diagnostics, Mannheim, Germany). Between December, 2012, and May, 2013, HPV testing was done in Colombia on 872 samples stored on the digene Specimen Transport Medium.

#### Colposcopy

According to the 2011 International Federation for Cervical Pathology and Colposcopy (IFCPC) classification, colposcopic examination included a general assessment and colposcopy findings. The general assessment measured adequacy, squamocolumnar junction visibility (fully visible, partially visible, and non-visible), and transformation zone (TZ) types 1–3. Colposcopy findings were classified as normal (normal colposcopic impression; including original squamous epithelium, Nabothian follicles, metaplastic squamous epithelium, and crypt or gland openings); positive minor or grade 1 (minor grade changes including fine vascular patterns, faint white epithelial uptake after acetic acid, irregular or geographical borders, and satellite lesions); positive major or grade 2 (major grade changes including sharp lesion borders, inner borders [within the TZ], presence of the ridge sign, dense or rapid uptake of acetic acid [or both], coarse vascular patterns [mosaic or punctate], and cuffed crypt or gland openings); or suspected invasive cancer (atypical vessels, fragile vessels, an irregular epithelial surface, exophytic lesions, necrosis, ulceration, tumour formation, or gross neoplasm).

At least one experienced colposcopist was selected per study centre, preferably with a clinical practice in the recruitment area, and was involved in extensive training sessions on study procedures and clinical management with detailed algorithms, continuous supervision, and quality assessment. Three of 15 colposcopists were already IFCPC colposcopy certified trainers, seven were trained to obtain the IFCPC colposcopy certificate, and two became certified trainers. At colposcopy, two to three biopsies from observed lesions were collected for all participants in whom acetowhite lesions were present and if the colposcopy was positive with TZ1 or TZ2. Endocervical sampling using cytology or histology (using the cell block technique) was recommended when a TZ3 was observed, and excision type 2 or 3 was performed when high-grade disease was diagnosed (HSIL+ cytology or CIN2+ histology).

#### Colposcopy-guided clinical management of participants

**Figure 1:**
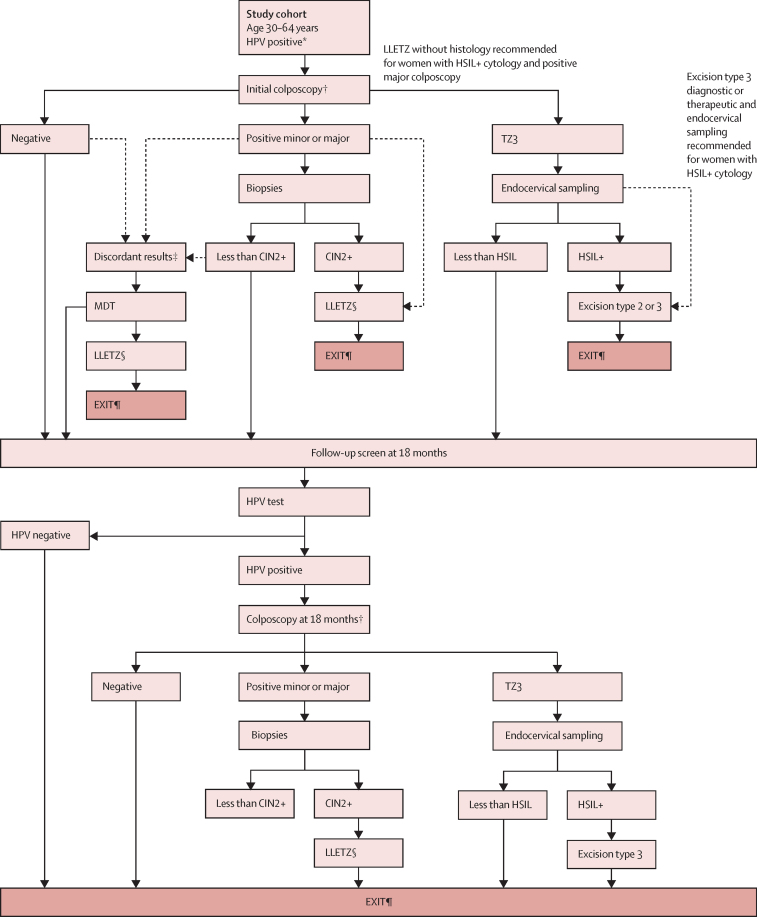
Colposcopy protocol Adapted from Almonte et al.^16^ CIN=cervical intraepithelial neoplasia. HPV=human papillomavirus. HSIL=high-grade squamous intraepithelial lesion. LLETZ=large loop excision of the transformation zone. MDT=multidisciplinary team. TZ=transformation zone. *Including HPV-negative women with abnormal cytology. †Evidence of invasive carcinoma led to biopsy and a referral. ‡Including HSIL+ cytology and negative colposcopy; HSIL+ cytology, positive minor colposcopy, and less than CIN2 biopsy; and less than HSIL cytology, positive major colposcopy, and less than CIN2 biopsy. §LLETZ type 1, 2, or 3. ¶EXIT indicates that the participants completed disease ascertainment and treatment as needed and returned to regular care.

The clinical management of HPV-positive women attending colposcopy was defined by the cytology and colposcopy result at enrolment (figure 1). A multidisciplinary team meeting was convened for each woman with discordant results defined as a normal colposcopy and HSIL+ cytology; or positive minor colposcopy, HSIL+ cytology, and less than CIN2 histology; or positive major colposcopy, less than HSIL cytology, and less than CIN2 histology. Multidisciplinary teams included at least the local principal investigator, colposcopist, cytopathologist, and pathologist and met to review all diagnoses, the age, and parity to decide between either treatment with large loop excision of the TZ (LLETZ) or recall at 18 months. To reduce the risk of women withdrawing from the study with untreated disease, LLETZ was prioritised rather than recall at 18 months; LLETZ was recommended for those with positive major colposcopy and high-grade cytology, when possible, without waiting for histological confirmation; and diagnostic or therapeutic excision type 3 was recommended for women with a TZ3 and HSIL+ cytology, without waiting for endocervical sampling.

At 18 months, the clinical management was guided by results of the second colposcopy (cytology was not repeated). Women with CIN2+ on local histology received excisional treatment as needed, exited the study, and returned to routine health care after treatment. Women with less than CIN2 on histology or less than HSIL on endocervical cytology or normal colposcopy also exited the study and returned to routine health care with specific indications for follow-up, offered by the same clinician.

#### Pathology

Cervical tissues collected by biopsy or LLETZ were fixed in buffered formalin at the colposcopy clinic. Local pathologists interpreted haematoxylin and eosin-stained slides and reported histology results using CIN classification (negative, CIN1, CIN2, CIN3, adenocarcinoma in situ, and invasive cancer). When there was not enough tissue or other deficiencies were present in the tissue, pathology was reported as inadequate. Additionally, all study histology is being reviewed by an external panel of international experts on cervical pathology. Diagnoses are reported with Lower Anogenital Squamous Terminology^17^ after using p16 immunohistochemistry, when indicated, to more accurately define histological HSIL (including p16-positive CIN2, CIN3, and adenocarcinoma in situ).

### Outcomes

Diagnostic accuracy of colposcopy was assessed to predict cervical precancer or cancer, considering a positive test result when the colposcopic impression at the initial colposcopy was positive minor, positive major, or suspected cancer, and considered negative otherwise (ie, normal). The main study outcome was histologically confirmed CIN3+ (defined as grade 3 or worse) detected at the initial visit or 18-month visit. The secondary study outcome was CIN2+ (defined as grade 2 or worse) detected at the initial visit or 18-month visit. Sensitivity for CIN3+, specificity for less than CIN2, and positive predictive value for CIN3+ were computed to assess the accuracy of colposcopy as primary results and sensitivity for CIN2+ and specificity for less than CIN3 were computed as secondary results.

### Statistical analysis

Sensitivity, specificity, and positive predictive value with 95% CIs were estimated to assess the performance of enrolment colposcopy. Logistic regression models were used to obtain estimates of performance indicators (overall and by age [30–49 *vs* 50–65 years], cytological results [negative, ASC-US or LSIL, or HSIL], type of TZ, and the number of biopsies). As a supplementary result, χ^2^ tests were used to compare cytological results at enrolment in women with a normal colposcopy versus those with negative or CIN1 histology and determine the association between the type of TZ and age. Mixed effects logistic regression models were also used to obtain an overall pooled estimate of performance indicators stratified by study centres and assess heterogeneity (visualised using a forest plot), considering variation between study centres as a random effect. Analyses were performed using R (version 4.1). The lme4 package was used to fit mixed effects logistic models.

### Role of the funding source

The funders of the study had no role in study design, data collection, data analysis, data interpretation, or writing of the report.

## Results

**Figure 2:**
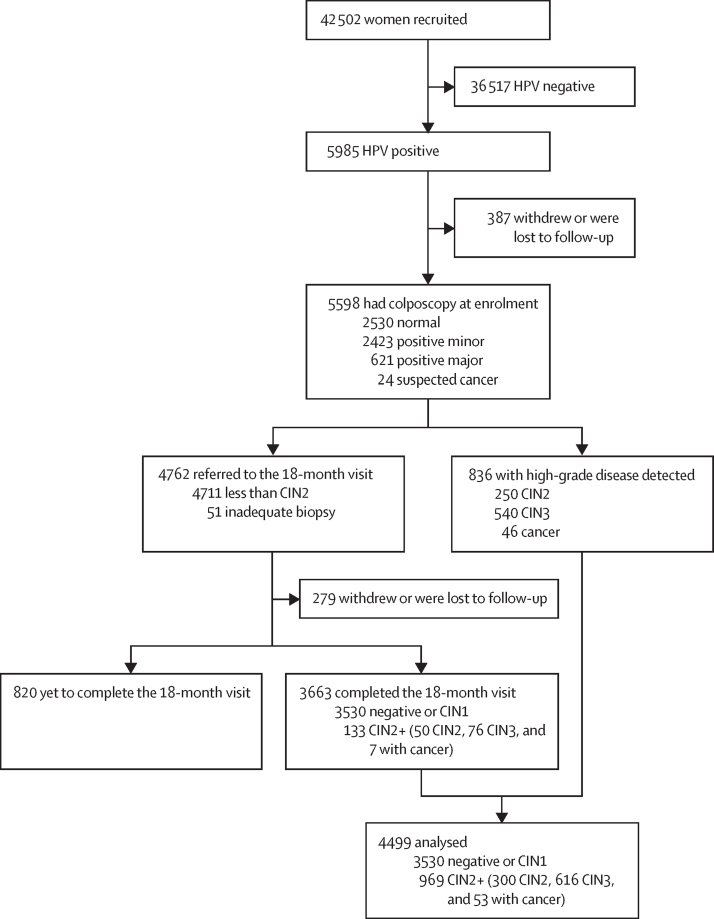
Study profile 4499 with complete follow-up included in the analysis. CIN=cervical intraepithelial neoplasia.

Between Dec 12, 2012, and Dec 3, 2021, 42 502 women were recruited and 36 517 (85·9%) were not included because they tested negative for HPV (figure 2). 5985 (14·1%) women tested positive for HPV and were referred to colposcopy. Of those, 5598 (93·5%) attended and completed the initial colposcopy with a median time of 1·8 months (IQR 1·2–2·8) after recruitment. 4762 (85·1%) of 5598 women with an initial normal colposcopy or with negative or CIN1 histology (less than CIN2 or inadequate biopsy) were recalled at 18 months for a second HPV test and referred to colposcopy (if positive). 279 (5·9%) of 4762 women who withdrew or were lost to follow-up and 820 (17·2%) who have yet to complete the 18-month follow-up (pending HPV test, colposcopy, or histology) were excluded. 4499 HPV-positive women with complete disease ascertainment were included in the analysis (969 [21·5%] with precancer or cancer [CIN2+], detected at enrolment [n=836] or at the 18-month visit [n=133]; 3530 [78·5%] negative or CIN1; figure 2).

Participant characteristics are shown in table 1. The median age of women was 40·6 years (IQR 34·7–49·9). Most women (4490 [99·8%] of 4499) had at least one previous cytology before recruitment, 798 (17·7%) had abnormal cytology at enrolment, and 240 (5·3%) had HSIL+. At the initial colposcopy, 2636 (58·6%) women had a positive colposcopic impression.

**Table 1:** Characteristics of HPV-positive women

		**Women (n=4499)**
Age, years		
	Median	40·6 (34·7–49·9)
	30–49	3378 (75·1%)
	50–65	1121 (24·9%)
Ever had a cytology		4490 (99·8%)
Cytology result at enrolment		
	Missing	130 (2·9%)
	Inadequate	69 (1·5%)
	Negative	3502 (77·8%)
	ASC-US	266 (5·9%)
	LSIL	292 (6·5%)
	HSIL+	240 (5·3%)
Initial colposcopy (colposcopic impression)		
	Normal	1863 (41·4%)
	Positive minor	2040 (45·3%)
	Positive major	574 (12·8%)
	Suspicion of cancer	22 (0·5%)
TZ type		
	TZ1	2288 (50·9%)
	TZ2	1039 (23·1%)
	TZ3	1172 (26·1%)
Number of biopsies		
	0	1759 (39·1%)
	1	525 (11·7%)
	2	1348 (30·0%)
	3	602 (13·4%)
	4	265 (5·9%)
Disease outcomes*		
	Negative†	2239 (49·8%)
	CIN1	1291 (28·7%)
	CIN2	300 (6·7%)
	CIN3‡	616 (13·7%)
	Cancer	53 (1·2%)

Data are median (IQR) or n (%). ASC-US=atypical squamous cells of undetermined significance. CIN=cervical intraepithelial neoplasia. HSIL=high-grade squamous intraepithelial lesion. LSIL=low-grade squamous intraepithelial lesion. TZ=transformation zone.

* As detected at the initial or 18-month visit (for participants with less than CIN2 at the initial visit).

† Includes histologically confirmed negative or normal colposcopy without biopsy at enrolment and at the 18-month follow-up visit.

‡ Includes adenocarcinoma in situ.

Overall, CIN3+ was detected in 669 (14·9%) of 4499 women at the initial visit or 18-month visit (616 [13·7%] with CIN3 and 53 [1·2%] with cancer). 2674 (75·8%) of 3530 women considered negative for disease (<CIN2) had biopsies with negative results or CIN1 histology, whereas 856 (24·2%) had normal colposcopy with no biopsy (appendix p 3). The percentage of women with ASC-US+ cytology at enrolment was significantly higher in those that had biopsies with negative or CIN1 histology (360 [13·5%]) than in those with normal colposcopy and no biopsy (49 [5·7%]; p<0·0001; appendix p 3).

Enrolment colposcopy was normal in 93 (9·6%) of 969 histologically confirmed women with CIN2+ (34 with CIN2, 55 with CIN3, and four with cancer [false negatives]) and positive in 1760 (49·9%) of 3530 women with less than CIN2 (1557 with positive minor and 203 with positive major [false positives]; table 2). Thus, the accuracy of colposcopy was 58·8% (2646 of 4499 correctly identified participants; 876 with CIN2+ [true positives], and 1770 with less than CIN2 [true negatives]). Sensitivity was 91·2% (95% CI 88·9–93·2) for CIN3+ and 90·4% (88·4–92·2) for CIN2+. Specificity was 50·1% (48·5–51·8) for less than CIN2 and 47·1% (45·5–48·7) for less than CIN3. The positive predictive value for CIN3+ was 23·1% (21·6–24·8; table 3).

**Table 2:** Disease outcomes (negative, CIN1–3, or cancer) by colposcopic impression

	Negative*	**CIN1**	**CIN2**	**CIN3**†	**Cancer**	**Total**
Normal	1568	202	34	55	4	1863 (41·4%)
Positive minor	600	957	184	285	14	2040 (45·3%)
Positive major	71	132	82	271	18	574 (12·8%)
Suspected cancer	0	0	0	5	17	22 (0·5%)
Total	2239 (49·8%)	1291 (28·7%)	300 (6·7%)	616 (13·7%)	53 (1·2%)	4499 (100%)

Data are n or n (%). Disease outcomes as detected at the initial visit or at the 18-month visit (for those with less than CIN2 at the initial visit). CIN=cervical intraepithelial neoplasia.

* Includes histologically confirmed negative and normal colposcopy without biopsy at enrolment and at the 18-month follow-up visit.

† Includes adenocarcinoma in situ.

**Table 3:** Performance of colposcopy as triage for HPV-positive women

		**True positive**	**False positive**	**False negative**	**True negative**	**Estimate (95% CI) or p value**
**All women (overall)**						
Sensitivity for CIN3+		610	2026	59	1804	91·2% (88·9–93·2)
Specificity for less than CIN3		..	..	..	..	47·1% (45·5–48·7)
Positive predictive value for CIN3+		..	..	..	..	23·1% (21·6–24·8)
Sensitivity for CIN2+		876	1760	93	1770	90·4% (88·4–92·2)
Specificity for less than CIN2		..	..	..	..	50·1% (48·5–51·8)
**By age**						
Sensitivity for CIN3+		..	..	..	..	p<0·0001
	30–49 years	534	1616	37	1191	93·5% (91·3–95·3)
	50–65 years	76	410	22	613	77·6% (68·6–85·0)
Specificity for less than CIN2		..	..	..	..	p<0·0001
	30–49 years	763	1387	61	1167	45·7% (43·8–47·6)
	50–65 years	113	373	32	603	61·8% (58·7–64·8)
**By cytology at screening**						
Sensitivity for CIN3+		..	..	..	..	p<0·0001
	Negative	287	1556	47	1612	85·9% (81·9–89·4)
	ASC-US or LSIL	138	317	4	99	97·2% (93·6–99·1)
	HSIL+	162	63	7	8	95·9% (92·1–98·2)
Specificity for less than CIN2		..	..	..	..	p_trend_<0·0001
	Negative	464	1379	76	1583	53·4% (51·6–55·2)
	ASC-US or LSIL	201	254	7	96	27·4% (22·9–32·3)
	HSIL+	174	51	7	8	13·6% (6·4–23·8)
**By TZ type**						
Sensitivity for CIN3+		..	..	..	..	p<0·0001
	TZ1	363	1213	24	688	93·8% (91·1–95·9)
	TZ2	175	485	12	367	93·6% (89·5–96·5)
	TZ3	72	328	23	749	75·8% (66·6–83·7)
Specificity for less than CIN2		..	..	..	..	p_trend_<0·0001
	TZ1	548	1028	41	671	39·5% (37·2–41·8)
	TZ2	230	430	21	358	45·4% (42·0–48·9)
	TZ3	98	302	31	741	71·0% (68·2–73·7)
**By the number of biopsies**						
Sensitivity for CIN3+		..	..	..	..	p_trend_=0·10
	0	45	42	53	1619	45·9% (36·3–55·8)
	1	53	414	2	56	96·4% (89·2–99·4)
	2	278	959	4	107	98·6% (96·7–99·6)
	3	160	421	0	21	100%
	4	74	190	0	1	100%
Sensitivity for CIN3+		..	..	..	..	p=0·10
	1–2	331	1373	6	163	98·2% (96·4–99·3)
	3–4	234	611	0	22	100%
Specificity for less than CIN2		..	..	..	..	p_trend_<0·0001*
	0	49	38	77	1595	97·7% (96·9–98·3)
	1	89	378	5	53	12·3% (9·4–15·6)
	2	411	826	10	101	10·9% (9·0–13·0)
	3	209	372	1	20	5·1% (3·2–7·6)
	4	118	146	0	1	0·7% (0·0–3·0)
Specificity for less than CIN2		..	..	..	..	p<0·0001
	1–2	500	1204	15	154	11·3% (9·7–13·1)
	3–4	327	518	1	21	3·9% (2·5–5·8)

Data obtained from the cross table by combining test results (positive *vs* typical colposcopic impression at enrolment) and disease (present *vs* absent). ASC-US=atypical squamous cells of undetermined significance. CIN=cervical intraepithelial neoplasia. HSIL=high-grade squamous intraepithelial lesion. LSIL=low-grade squamous intraepithelial lesion. TZ=transformation zone.

* p value computed to assess trend from one to four biopsies (excluding women with colposcopy but no biopsy).

**Figure 3:**
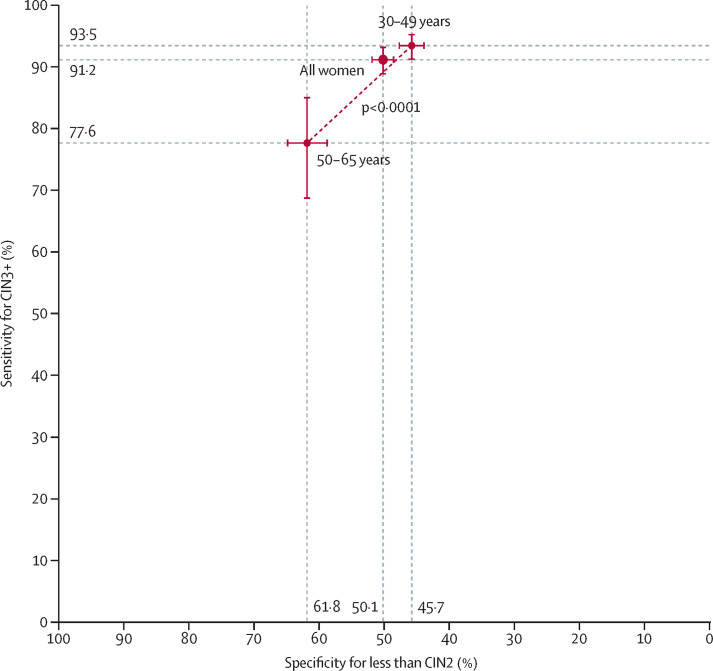
Performance of colposcopy to detect CIN3+ in HPV-positive women Bars show 95% CIs. Estimates are shown overall and stratified by age group (30–49 years *vs* 50–64 years). The dotted line represents the decrease in sensitivity and increase in specificity associated with age. Statistically significant differences between age groups were found for sensitivity and specificity (p<0·0001 for both). CIN=cervical intraepithelial neoplasia. HPV=human papillomavirus.

The sensitivity for CIN3+ significantly decreased in older women (93·5% [95% CI 91·3–95·3] in those aged 30–49 years *vs* 77·6% [68·6–85·0] in those aged 50–65 years; p<0·0001), whereas the specificity for less than CIN2 significantly increased with age (45·7% [43·8–47·6] *vs* 61·8% [58·7–64·8]; p<0·0001; table 3; figure 3). The sensitivity for CIN3+ was also significantly lower in women with negative cytology (85·9% [81·9–89·4]) than in those with abnormal cytology (97·2% [93·6–99·1] for ASC-US or LSIL and 95·9% [92·1–98·2] for HSIL+; p<0·0001). A significantly decreasing specificity for less than CIN2 was observed by cytological grade (53·4% [51·6–55·2] for negative cytology, 27·4% [22·9–32·3] for ASC-US or LSIL, and 13·6% [6·4–23·8] for HSIL+; p_trend_<0·0001; table 3). Furthermore, colposcopy was less accurate in detecting disease among 1172 (26·1%) women with TZ3 (sensitivity for CIN3+ significantly decreased to 75·8% [66·6–83·7]; p<0·0001; Table 1, Table 3). TZ3 was also associated with older age (660 [58·9%] of 1121 women aged 50–65 years *vs* 512 [15·1%] of 3378 women aged 30–49 years; p<0·0001; appendix p 4). Additionally, sensitivity was excellent regardless of the number of biopsies collected (98·2% [96·4–99·3] for one to two biopsies and 100% for three to four biopsies; p=0·10; table 3).

Both the sensitivity (range 67–100%) and specificity (29–81%) of colposcopy were highly variable across study centres. However, pooled estimates were similar to unadjusted overall estimates, but with notably wider CIs (appendix p 2).

## Discussion

To our knowledge this is the first large evaluation of colposcopy as triage for HPV-positive women. Our main result is the excellent performance of colposcopy with high overall sensitivity (91·2%) for CIN3+ detection in 4499 HPV-positive women (90·4% for CIN2+ detection), a high positive predictive value (23·1%) compared with the proportion of CIN3+ detected among all HPV-positive women (14·9%), and an acceptable specificity for less than CIN2 (50·1%) in the context of triage. Notably, colposcopy performance in terms of sensitivity was lower in women aged 50–65 years than in those aged 30–49 years and higher with abnormal cytology than in those with negative cytology (data unmasked to the colposcopists).

In the past 30 years, colposcopy accuracy has mainly been studied in women with abnormal cytology, showing highly variable sensitivity estimates (range 30–100%), possibly due to differences in positivity thresholds, disease definitions, and methods used for evaluation.^8^, ^9^, ^11^, ^12^, ^13^, ^14^, ^18^, ^19^, ^20^, ^21^, ^22^, ^23^, ^24^, ^25^ Additionally, training and expertise of colposcopists, the number of biopsies collected, and where in the ectocervix to collect the biopsies or endocervical samples (for TZ2 or TZ3) are factors that can substantially influence the detection and treatment of CIN2+ lesions. Furthermore, a meta-analysis^26^ performed for current WHO guidelines^6^ was the first to evaluate the accuracy of colposcopy specifically as triage in HPV-positive women. This analysis used individual data from three previous studies on cervical cancer screening, had a small sample size, and reported a sensitivity of 86% (95% CI 78–92) and specificity of 72% (61–83) for the performance of colposcopy to predict CIN3+.

As confirmed in a previous study,^8^ but also as a natural consequence of performing serial tests (ie, first HPV testing and then colposcopy), higher sensitivity of colposcopy is to be expected when used as triage in HPV-positive women rather than as primary screening because triage selects patients at a higher risk, so those with the disease are more likely to have a positive test, thus increasing the sensitivity. Nonetheless, the high sensitivity observed in our study also reflects ESTAMPA efforts to maximise disease detection through high-quality and standardised colposcopy, which was achieved using a clinical management protocol validated by international experts; collection of two to three biopsies from lesions and endocervical sampling for TZ3; streamlining of clinical management using cytology and colposcopy results; regular training on the colposcopy study protocol and meetings with colposcopists; and monitoring of HPV and disease prevalence over time to provide refresher training and apply corrective measures when needed. Despite all our efforts, heterogeneity of colposcopy performance was high across study centres, possibly because of the inherent subjectivity of colposcopy, previous colposcopy experience, and differences in the availability of adequate equipment (specifications, calibration, and maintenance) and medical supplies. However, the results reported here represent a precise pooled estimate of the performance of high-quality standardised colposcopy independently of these contextual factors.

Importantly, colposcopy performed less well in women aged 50–65 years, which highlights the difficulties of visualising the squamocolumnar junction in older women for biological reasons (eg, menopause and hormone-related factors) and re-emphasises the need to sample the endocervical canal. However, adequate training on endocervical sampling needs to be provided as the subjectivity of colposcopy, which typically focuses on evaluation of the ectocervix, increases when attempting to evaluate the endocervical canal. Furthermore, the sensitivity of colposcopy decreased in women with TZ3, which was also associated with older age. Moreover, we did not find a significant association of the sensitivity of colposcopy with the number of biopsies. The decision on the number of biopsies collected was made individually for each woman by colposcopists, who had the recommendation of collecting two to three biopsies in the presence of acetowhite lesions but might have decided to collect fewer biopsies in the more obvious lesions. Further analyses exploring the association between colposcopy performance and type of TZ, endocervical sampling, the number of biopsies, type of lesion, colposcopist experience, and HPV genotypes (notably HPV16) overall and stratified by age will be reported separately.

The main strength of our study is the large number of colposcopies performed with high participant compliance (>90% colposcopy attendance in all study centres) and done by sufficiently equipped, trained, and supervised colposcopists, which allowed the detection and treatment of 969 (21·5%) of 4499 women with confirmed CIN2+. Additionally, the study was not severely affected by the COVID-19 pandemic because 3873 (86·1%) women included in this analysis completed participation before the pandemic started.

We noted three main limitations in our study. First, biopsies were not collected from women with normal colposcopy (as recommended by current clinical guidelines^27^), which could have induced verification bias when evaluating the performance of the initial colposcopy. However, the study design included a second screening at 18 months to ensure that disease not detected on initial screening was adequately treated. At this visit, women with initial normal colposcopy or CIN1 had another HPV test, and those positive for HPV had another colposcopy and biopsy, thus reducing the likelihood of verification bias. Nevertheless, the absence of disease for participants attending this 18-month follow-up visit who had an initial negative colposcopy was assumed (ie, not verified) if they tested negative for HPV or had normal colposcopy with no biopsy (and exited the study afterwards). Moreover, the presence of lesions was further assessed in women who had endocervical sampling at the initial or 18-month visit, which also reduced verification bias when no biopsies were taken. For clinical safety of participants, any woman who withdrew from the study was referred to their health providers with our findings and specific indications for follow-up, which was usually performed by the same study colposcopist. Under this rationale, 1099 women with less than CIN2 at the initial visit who did not complete the 18-month visit were excluded from the performance analysis. Computations including these participants as free-of-disease for the main outcome would lead to the same sensitivity (same true positive and false negative frequencies), but slightly higher specificity (52·7%) and a decreased positive predictive value (19·8%; 432 additional false positives and 667 additional true negatives; data not shown). Similar colposcopic impression at enrolment was observed in participants lost to follow-up (140 [50·2%] positive colposcopies of 279) compared with those who completed the 18-month visit (1831 [50%] positive colposcopies of 3663; p=0·99), suggesting absence of bias. Second, performance of colposcopy was assessed in HPV-positive women, therefore mirroring a screen, triage, and treat approach in which HPV positivity (screen) is needed before a triage test (colposcopy) can be done to obtain a diagnostic and eventually treat the woman. However, colposcopists were aware of cytological results at enrolment and used this information together with the colposcopy results to guide clinical management of HPV-positive women, which could have increased the positivity of the colposcopic impression. We observed that performance of colposcopy improved with cytological grade, suggesting that knowing the cytology result could have influenced performance, particularly in relation to PPV. Even though ESTAMPA plans to evaluate several other triage tests (such as HPV genotyping, p16–ki67 dual-staining, E6–E7 mRNA detection, and methylation), the colposcopy protocol did not include consideration of such tests. Third, our colposcopy performance cannot be extrapolated to clinical settings unless a similar standard quality-assured protocol for HPV-positive women is followed. Yet, we show that in a high-quality clinical scenario, colposcopy could reach maximal disease detection, thus reinforcing the importance of clear clinical colposcopic guidelines and training.

We are completing a histology review that will adjudicate disease of all histological specimens by an international expert panel, who will report results using Lower Anogenital Squamous Terminology. Currently, 70% of local diagnoses have been reviewed showing similar colposcopy performance (92·5% sensitivity and 57·3% specificity for histological HSIL+).

Current WHO recommendations^6^ for cervical screening and treatment highlight colposcopy as a potential triage for HPV-positive women (along with partial HPV genotyping, visual inspection with acetic acid, or cytology). However, since HPV testing is replacing cytology as primary screening, high referral rates might require an increase in colposcopy capacity and monitoring systems to ensure acceptable follow-up rates of HPV-positive women. This point is particularly relevant when considering implementing colposcopy as first-line triage at a population level because in HPV screen-and-treat schemes, a proportion of HPV-positive women who are not eligible for ablative treatment will require colposcopic evaluation. Additionally, new colposcopy devices with computer-assisted imaging systems are being developed or evaluated and might become point-of-care triage for immediate treatment.

This multicentre study conducted across Latin America is the first to comprehensively evaluate the performance of different triage techniques to detect cervical precancer and cancer in HPV-positive women. ESTAMPA represents a great effort on standardisation of colposcopy in this heterogeneous region (in terms of access to health, health investment, policies, culture, and geography). We showed that colposcopy can be optimised with proper standardisation of the protocol to effectively triage HPV-positive women. Our findings not only directly contribute to improving the clinical management of HPV screen-positive women but might also lead to reinforcing the role of colposcopy in cervical cancer screening worldwide.

## Data sharing

The data used in this study selected several variables (age at recruitment, screening history, cytology results at screening, colposcopy, histology results at the initial and 18-month visit, and the type of transformation zone and number of biopsies at the initial visit) from the EStudio multicéntrico de TAMizaje y triaje de cáncer de cuello uterino con pruebas del virus del PApiloma humano (ESTAMPA) study database, which is stored securely at the International Agency for Research on Cancer. Anonymised individual participant data or aggregate data will be made available upon reasonable request to the corresponding author (vallsj@iarc.who.int), after signing a contract, and with approval from the principal and local investigators.

## Declaration of interests

GV reports conference fees from Roche. GIS reports provision of reagents from Roche. CP reports royalties and conferences fees from Roche, MSD, Seegene, Abbot, and Exeltis. TMD reports grants from the US National Cancer Institute, consulting fees from Antiva, and participation on an advisory board for Roche. All other authors declare no competing interests.
