# Supplementary material for: Performance of standardised colposcopy to detect cervical precancer and cancer for triage of women testing positive for human papillomavirus: results from the ESTAMPA multicentric screening study
Source: Lancet Glob Health. 2023 Feb 14;11(3):e350–60. doi: 10.1016/S2214-109X(22)00545-9 (PMC10020136; doi:10.1016/S2214-109X(22)00545-9)
Supplement: Supplementary appendix [file mmc1.pdf]

THE LANCET
Global Health

Supplementary appendix

This appendix formed part of the original submission and has been peer reviewed. We post it as supplied by the authors.

Supplement to: Valls J, Baena A, Venegas G, et al. Performance of standardised colposcopy to detect cervical precancer and cancer for triage of women testing positive for human papillomavirus: results from the ESTAMPA multicentric screening study. *Lancet Glob Health* 2023; **11:** e350–60.

Supplementary Material for

Performance of standardised colposcopy to detect cervical precancer and cancer for triage in women positive for human papillomavirus: results from the ESTAMPA multicentre, cross-sectional, screening study

Joan Valls, Armando Baena, Gino Venegas, Marcela Celis, Mauricio González, Carlos Sosa, Jorge Luis Santin, Marina Ortega, Ana Soilán, Elmer Turcios, Jacqueline Figueroa, Margarita Rodríguez de la Peña, Alicia Figueredo, Andrea Verónica Beracochea, Natalia Pérez, Josefina Martínez-Better, Oscar Lora, Julio Yamil Jiménez, Diana Giménez, Laura Fleider, Yuly Salgado, Sandra Martínez, Yenny Bellido-Fuentes, Bettsy Flores, Silvio Tatti, Verónica Villagra, Aurelio Cruz-Valdez, Carolina Terán, Gloria Inés Sánchez, Guillermo Rodríguez, Maria Alejandra Picconi, Annabelle Ferrera, Laura Mendoza, Alejandro Calderón, Raul Murillo, Carolina Wiesner, Nathalie Broutet, Silvana Luciani, Carlos Pérez, Teresa M Darragh, José Jerónimo, Rolando Herrero, Maribel Almonte, on behalf of the ESTAMPA study group

Correspondence to:

Dr Joan Valls, Early Detection, Prevention and Infections Branch, International Agency for Research on Cancer, Lyon 69366, France

e-mail: vallsj@iarc.who.intfr

Contents

Figure S1. Performance forest plot………………………………………………………………. 2
Table S1. Cytology results at enrolment in HPV-positive women considered negative for disease.. 3
Table S2. Type of transformation zone by age groups among HPV-positive analysed participants.. 4
Additional members of the ESTAMPA study group…………………………………………….. 5

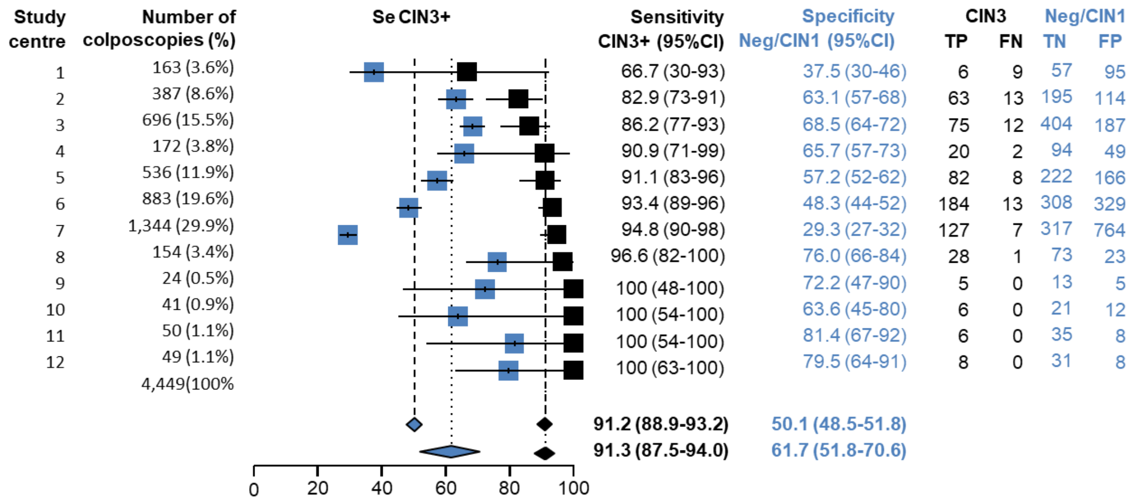

Figure S1: *Performance forest plot*. Individual estimates for sensitivities for CIN3+ (black) and specificities excluding CIN2 (blue) for each ESTAMPA study centre and pooled overall estimates (with fixed or random effects model, accounting for study centre heterogeneity). Study centres are ordered according to their sensitivity estimates. Total number of colposcopies, frequency of true positives (TP) and false negatives (FN), for sensitivity calculations, and true negatives (TN) and false positive, for specificity calculations excluding CIN2 cases, are shown.

Cytology at enrolment	Women considered negative for disease n=3,530		p-value
	**Normal colposcopy n=856**	**Negative or CIN1 histology n=2,674**	
Insufficient or missing	41 (4.8%)	118 (4.4%)	0.71
Negative	766 (89.5%)	2,196 (82.1%)	<0.001
ASC-US	24 (2.8%)	158 (5.9%)	<0.001
LSIL	22 (2.6%)	146 (5.5%)	<0.001
HSIL	3 (0.3%)	56 (2.1%)	<0.001
	856 (100%)	2,674 (100%)	
ASC-US+	49 (5.7%)	360 (13.5%)	<0.0001

Table S1: *Cytology results at enrolment in HPV-positive women considered negative for disease (with normal colposcopy with no biopsies or with negative or CIN1 histology)*. Absolute frequency (and percentage) of cytological results shown. P-values from chi-square tests assessing the differences observed in the percentages for each cytological grade and grouping ASC-US, LSIL and HSIL (ASC-US+) shown.

	Age		All women
	**30-49 years n=3,378 (75·1%)**	**50-64 years n=1,121 (24·9%)**	**n=4,499 (100%)**
Type of transformation zone			
Type 1	2104 (62·3%)	184 (16·4%)	2,288 (50·9%)
Type 2	762 (22·6%)	277 (24·7%)	1,039 (23·1%)
Type 3	512 (15·1%)	660 (58·9%)	1,172 (26·1%)
	Chi-squared test p<0.0001		

Table S2: Type of transformation zone by age groups among HPV-positive analysed participants. Absolute frequency (and percentage) shown. P-value from a chi-squared test assessing the association of type of transformation zone with age groups shown.

Additional members of the ESTAMPA study group

Eduardo Lazcano-Ponce, Instituto de Salud Pública de México, Morelos, México.

Catterina Ferreccio, Advanced Center for Chronic Diseases, ACCDiS, Facultad de Medicina, Pontificia Universidad Católica de Chile, Santiago, Chile.

Mary Luz Rol, Eric Lucas, Arianis Tatiana Ramírez, Early Detection, Prevention and Infections Branch, International Agency for Research on Cancer, Lyon.

María de la Luz Hernández, SMS-Oncology, Amsterdam, The Netherlands.

María Isabel Rodríguez , Instituto de Investigaciones en Ciencias de la Salud, Universidad Nacional de Asunción, San Lorenzo, Paraguay.

Franco Doimi, Patología Oncológica SAC, Lima, Perú.

Yessy Cabrera, Instituto de Investigaciones en Microbiología, Universidad Nacional Autónoma de Honduras (UNAH), Tegucigalpa, Honduras.

Brenda Salgado, Secretaría de Salud, Tegucigalpa, Honduras.

Laura García, Laboratorio de Biología Molecular, Departamento de Patología Clínica, Centro Hospitalario Pereira Rossell, Montevideo, Uruguay.

María Celeste Colucci, Instituto Nacional de Enfermedades Infecciosas - ANLIS Malbrán, Buenos Aires, Argentina.

Benedicta Caserta, Departamento de Anatomía Patológica y Citología, Hospital de la Mujer, Centro Hospitalario Pereira Rossell, Montevideo, Uruguay.
